# circAtlas 3.0: a gateway to 3 million curated vertebrate circular RNAs based on a standardized nomenclature scheme

**DOI:** 10.1093/nar/gkad770

**Published:** 2023-09-22

**Authors:** Wanying Wu, Fangqing Zhao, Jinyang Zhang

**Affiliations:** Beijing Institutes of Life Science, Chinese Academy of Sciences, Beijing 100101, China; Beijing Institutes of Life Science, Chinese Academy of Sciences, Beijing 100101, China; University of Chinese Academy of Sciences, Beijing 100049, China; Key Laboratory of Systems Biology, Hangzhou Institute for Advanced Study, University of Chinese Academy of Sciences, Chinese Academy of Sciences, Hangzhou, China; Beijing Institutes of Life Science, Chinese Academy of Sciences, Beijing 100101, China

## Abstract

Recent studies have demonstrated the important regulatory role of circRNAs, but an in-depth understanding of the comprehensive landscape of circRNAs across various species still remains unexplored. The current circRNA databases are often species-restricted or based on outdated datasets. To address this challenge, we have developed the circAtlas 3.0 database, which contains a rich collection of 2674 circRNA sequencing datasets, curated to delineate the landscape of circRNAs within 33 distinct tissues spanning 10 vertebrate species. Notably, circAtlas 3.0 represents a substantial advancement over its precursor, circAtlas 2.0, with the number of cataloged circRNAs escalating from 1 007 087 to 3 179 560, with 2 527 528 of them being reconstructed into full-length isoforms. circAtlas 3.0 also introduces several notable enhancements, including: (i) integration of both Illumina and Nanopore sequencing datasets to detect circRNAs of extended lengths; (ii) employment of a standardized nomenclature scheme for circRNAs, providing information of the host gene and full-length circular exons; (iii) inclusion of clinical cancer samples to explore the biological function of circRNAs within the context of cancer and (iv) links to other useful resources to enable user-friendly analysis of target circRNAs. The updated circAtlas 3.0 provides an important platform for exploring the evolution and biological implications of vertebrate circRNAs, and is freely available at http://circatlas.biols.ac.cn and https://ngdc.cncb.ac.cn/circatlas.

## Graphical Abstract

**Graphical Abstract:**
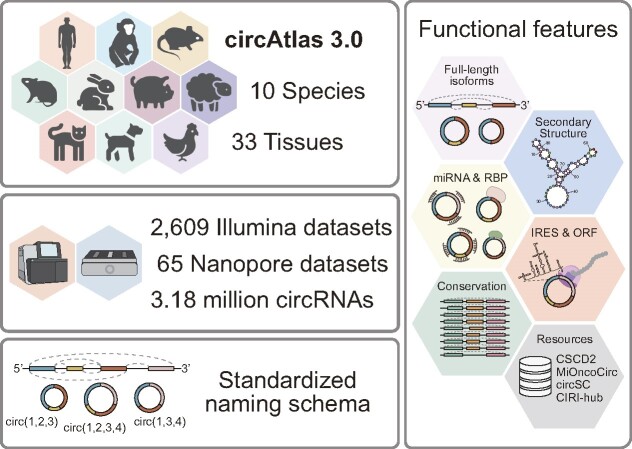

## Introduction

Circular RNAs (circRNAs) are a large class of covalently closed circular transcripts that are widely existed in eukaryotes. Recent studies have demonstrated the important regulatory role of circRNAs, including miRNA/RBP sponge (1,2), encoding peptide (3,4), regulating transcription (5,6) and translation (7,8). Despite the increasing studies of circRNAs over the past decade, the overall landscape of circRNAs within vertebrates still remains significantly underexplored. Therefore, a comprehensive exploration of circRNAs across different species is essential to understand their functions in different biological processes.

The growing number of circRNA sequencing datasets has facilitated the emergence of multiple databases aimed at elucidating the circRNA landscape in different species (9–12). However, most of these databases are still limited to specific species or contain a relatively small number of datasets. In 2020, we developed the circAtlas 2.0 (13), which included a repository of one million circRNAs from six distinct species. Given the substantial expansion of circRNA sequencing datasets in recent years, a more comprehensive database of the vertebrate circRNA landscape and their functional profiles has become an imperative requirement. This urgency arises from the necessity to establish a robust platform that not only facilitates insights into circRNA evolution but also serves as a means to prioritize functionally significant circRNA candidates.

The high sequence similarity of circRNA and its cognate linear counterparts makes it difficult to reconstruct full-length circRNA isoforms using short-read Illumina RNA-seq (14). Although several computational tools have been developed to reconstruct circRNAs from these short-read sequencing data (15,16), the reconstruction of long circRNA sequences remains constrained (17). Recent advances in long-read sequencing technologies have promoted the development of several long-read based circRNA sequencing strategies (18–22). Using the rolling circle reverse transcription or RCA assay, these methods can reconstruct the full-length circRNA sequences, enabling the robust profiling of full-length circRNAs across extended genomic spans. Thus, these long-read sequencing datasets offer a distinct advantage in discerning circRNA internal structures and enabling effective functional prioritization.

To this end, we constructed the circAtlas 3.0 database, which comprises a comprehensive compendium of 2609 Illumina and 65 nanopore RNA-seq datasets from 33 diverse tissues within 10 distinct species. Compared to other resources, circAtlas 3.0 provides the largest collection of over 3 million vertebrate circRNAs, with their full-length sequences and comprehensive functional analyses including orthologous circRNAs, secondary structure, miRNA binding sites, RBP binding sites and IRES/ORF prediction. In addition, circAtlas 3.0 also incorporated a standardized nomenclature scheme for circRNAs, which will bridge the gap arising from inconsistent nomenclature practices across various circRNA resources.

## Materials and methods

### Data collection

To obtain a comprehensive cohort for profiling circRNA across different species, a total of 2609 Illumina and 65 nanopore RNA-seq datasets from 33 different tissues in 10 species were downloaded from the SRA (https://www.ncbi.nlm.nih.gov/sra) (23) and GSA (https://ngdc.cncb.ac.cn/gsa/) (24) databases. The hg38 and mm10 reference genomes and GTF annotations were downloaded from the GENCODE project (25), and other genomes of rheMac8, rn6, oryCun2, susScr11, oviAri3, felCat9, canFam3 and galGal4 were downloaded from the UCSC Genome Browser database (26). The summary of the newly collected samples compared to circAtlas 2.0 can be found in Supplementary Table S1.

### Identification and reconstruction of full-length circRNAs

For Illumina RNA-seq data, the cleaned reads were aligned to the reference genome using bwa (v0.7.17) (27), and circRNAs were subsequently identified and quantified using the CIRI2 (v2.0.6) (28,29) and CIRIquant (v1.1.2) (30) pipeline. To reconstruct the full-length sequence of circRNA isoforms, the CIRI-full (v2.1.1) (15,16,31) and CIRI-vis (v1.4.1) (32) pipeline were employed to assemble full-length circRNAs using the reverse overlap feature. To further reduce the number of false positives (33), three other software, CIRCexplorer2 (v2.3.8) (34), DCC (v0.5.0) (35) and find_circ (v1.2) (36), were also used to identify circRNAs, and circRNAs that are supported by at least one of the other tools were retained for further analysis.

**Figure 1.**
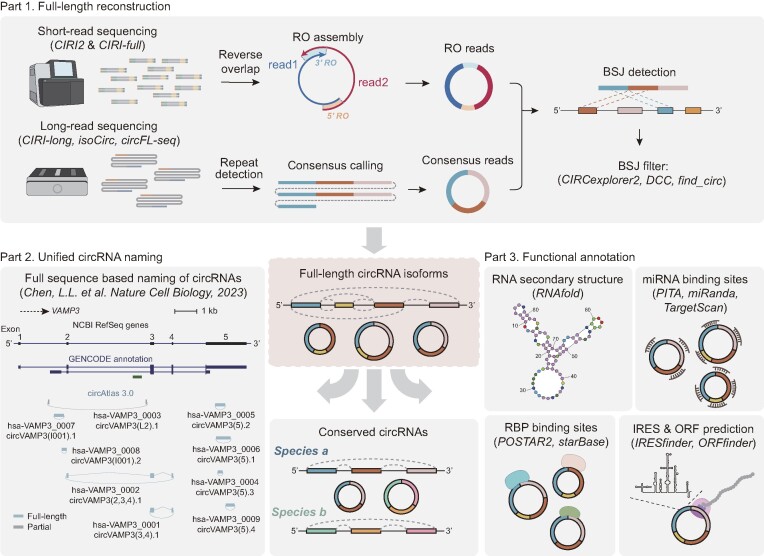
Schematic of the data processing pipeline in the circAtlas 3.0 database. The circAtlas 3.0 integrated both Illumina and nanopore sequencing datasets to identify full-length circRNA isoforms, and three additional circRNA identification tools were used to filter high-confidence back-splicing events. Based on the identified circRNA isoforms, the circRNAs were named according to the host gene and circular exon annotation. Then, orthology analysis and functional annotation including RNA secondary structure, miRNA/RBP binding sites and IRES/ORF prediction were performed as described in the Materials and Methods section.

For long-read sequencing data, the raw sequencing data from the CIRI-long protocol were trimmed using Porechop (https://github.com/rrwick/Porechop), and CIRI-long (v1.1.0) (18,37) was performed to identify circRNAs from nanopore sequencing reads. For other long-read circRNA sequencing protocols including isoCirc (19), circFL-seq (20) and an adapted version of CIRI-long (38,39), the processed data were obtained from their original manuscripts. Finally, the reconstructed full-length sequences were used to annotate the spliced isoforms of the identified circRNAs (Figure 1).

### Standardized naming of circRNAs

To achieve a standardized nomenclature scheme between different circRNA resources, we employed the circRNA naming criteria that recently proposed by the circRNA community (40). In brief, circRNAs were first divided into different types, and then named according to the order of their circular exons. The final unified ID of each circRNA consisted of the circRNA type, the name of their gene, the annotation of each circular exon, and an appended numerical ID to distinguish circRNAs with the same exon annotation, which provides nomenclature information on the internal structure of circRNAs.

### Orthology analysis of circRNAs

Orthologous circRNAs were predicted using a customized assay similar to circAtlas 2.0 (13). Briefly, the candidate orthologous genes were first obtained from the OMA orthology database (41). Then, the 50 bp fragments on both sides of the circRNA back-splicing junction (BSJ) were extracted to represent the BSJ sequence. Both full-length and BSJ sequences of circRNAs in in orthologous gene pairs were then aligned using BLAT (42) and a threshold of 50% identity of full-length or BSJ sequences. Finally, orthologous circRNA groups were determined using MultiMSOAR v2.0 software (43).

### Conversion of circRNA ID and position in different genome assemblies

circAtlas 3.0 provides user-friendly conversion of circAtlas ID to various databases including circBase (9), circRNADb (44), deepBase v2.0 (45), CIRCpedia v2 (10). The conversion of circRNA BSJ between circAtlas 3.0 and several commonly used assemblies (hg19, mm9, rheMac10, rn7, susScr3, oviAri4, felCat8, canFam6, galGal6) was performed using LiftOver.

### RNA structure and functional annotation

Based on the assembled full-length circRNA sequences, the secondary structure was predicted using the RNAfold algorithm of the Vienna RNA 2.0 package (46). To predict the circRNA-miRNA interaction, mature miRNA sequences of different species were downloaded from miRBase (47). Then, three independent miRNA prediction software, miRanda (v3.3.a) (48), TargetScan (v7.0) (49) and PITA (v2.1.2) (50), were used to predict the circRNA-microRNA binding position. The predicted miRNA response elements on the circRNAs were integrated to represent the possible circRNA-miRNA interactions. To predict the circRNA-RBP interaction, the high-confidence RBP binding sites were downloaded from the starBase (v2.0) (51) and POSTAR2 (52) database. The RBP binding sites located within the 1 kb flanking region of BSJs or within the circular exons were retained for further analysis. To explore the coding potential of circRNAs, IRESfinder (v1.1.0) (53) and ORFfinder (https://www.ncbi.nlm.nih.gov/orffinder) were performed to predict IRES and coding peptides in the circRNA sequence.

### Database deployment

The circAtlas 3.0 database was deployed in a virtual machine running CentOS 7.9. The backend of the circAtlas 3.0 database was based on MySQL (v14.14), and the web server was deployed using Apache (v2.4.6) and PHP (v7.4.33). The interactive visualization of the analysis results was implemented using jQuery (3.6.4), Apache ECharts (5.4.0) (54) and D3.js (v3.5.17). All online analysis was performed using Python (v3.10.4). The circAtlas 3.0 database is publicly available at http://circatlas.biols.ac.cn and https://ngdc.cncb.ac.cn/circatlas, and no login is required. Users can easily download the detected circRNAs and full-length circRNA sequences. All software involved in the circAtlas 3.0 is also listed on the download page.

## Results

### A comprehensive atlas of circRNAs across 10 vertebrate species

**Figure 2.**
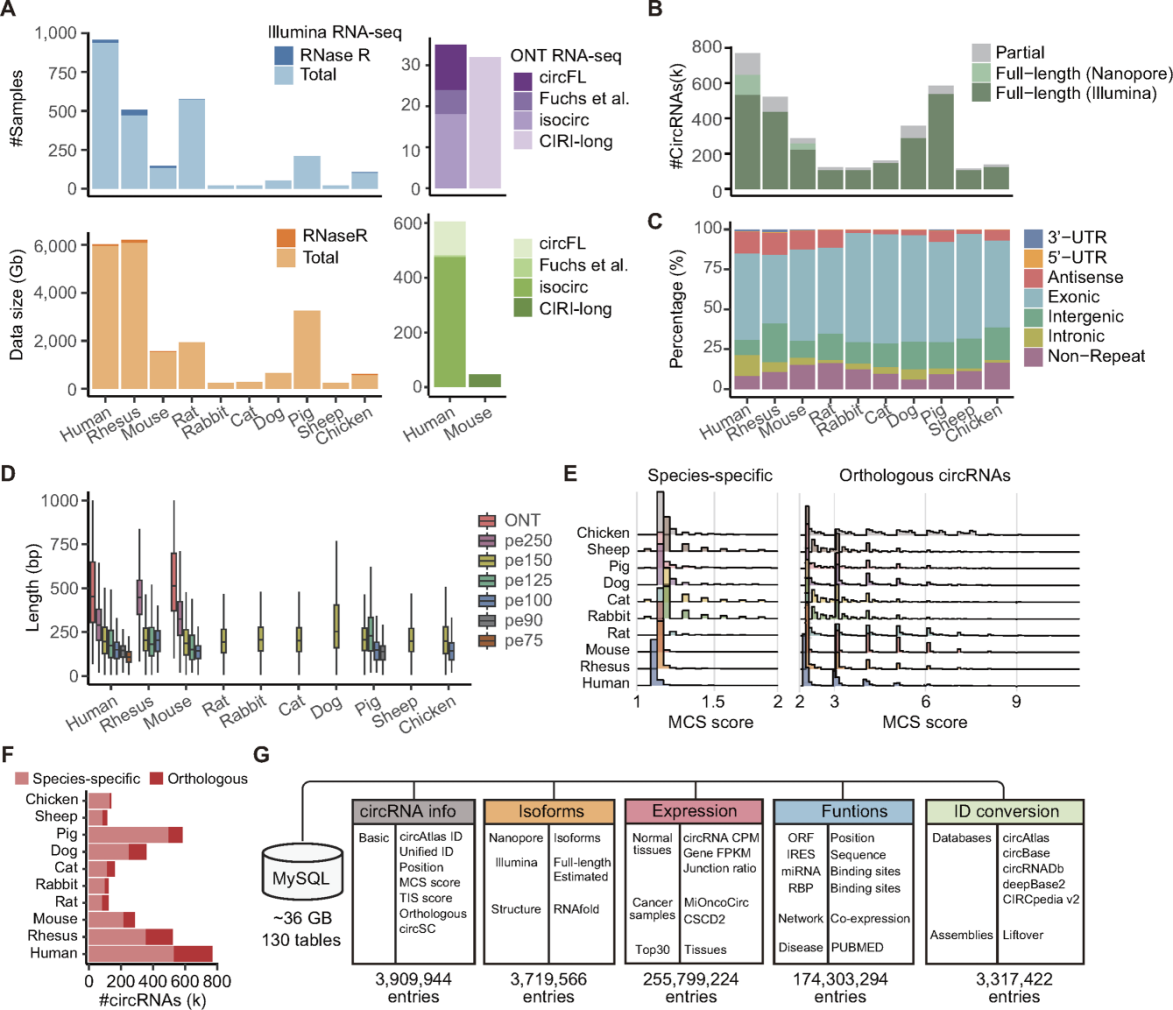
Data statistics and summary of identified circRNAs. (**A**) Number of collected samples and total library size in each species. The nanopore sequencing datasets were divided according to their sequencing protocol. (**B**) Number of partially assembled and reconstructed full-length circRNAs in each species. (**C**) Proportion of circRNAs in different genomic regions. (**D**) Length of reconstructed circRNA in each species. Colors represent circRNAs detected by different sequencing strategies. (**E**) The distribution of MCS scores of the detected circRNAs. All circRNAs were divided into species-specific circRNAs (left) and orthologous circRNAs that are conservatively expressed in multiple species (right). (**F**) Number of species-specific and orthologous circRNAs in each species. (**G**) Schematic view of the MySQL database structure of circAtlas 3.0.

The circAtlas 3.0 contains an updated compendium of 2536 total RNA and 73 RNase R treated Illumina RNA-seq libraries from 10 vertebrate species. In order to achieve reliable full-length circRNA isoform reconstruction, we have also included 65 long-read circRNA sequencing data from human and mouse samples. In total, 21.02 terabytes of Illumina and 651.62 gigabytes of Nanopore sequencing data were collected, forming a rich resource for profiling circRNAs across different species (Figure 2A). Through stringent filters, we identified over 3.18 million circRNAs across 10 species, with a remarkable 79.49% of these circRNAs assembled into full-length sequences. Notably, 15.06% of human and 13.23% of mouse circRNAs in our dataset were supported by nanopore long reads, significantly enhancing the foundational support for downstream functional analyses (Figure 2B).

To better demonstrate the diversity of circRNAs, all circRNAs were divided into different groups according to the region of their back-splicing junctions (Figure 2C). Interestingly, we observed a significantly high increase in the proportion of antisense circRNAs from carnivores to primates, which is consistent with the pervasive antisense transcription of mammalian genes (55). It should be noted that ciRS-7, the most famous circRNA that was first found to be a miRNA sponge (56), also originates from the antisense strand of the CDR1 gene. Considering that these antisense circRNAs may have a potential function to bind to cognate precursor mRNAs, these findings underscore the utility of circAtlas 3.0 to explore the regulatory roles of vertebrate circRNAs. As shown in Figure 2D, the average length of the reconstructed circRNAs ranged from 200 to 500 nucleotides across all 10 species. Since different read lengths can affect the ability to reconstruct longer circRNAs (16), the length of circRNAs detected by different sequencing strategies was calculated separately. Here, a dramatic increase in circRNA length was observed with the increasing sequencing length. The incorporation of nanopore sequencing datasets substantially broadens the scope of circRNA detection, magnifying the range from the 200–300 nt observed in Illumina RNA-seq to an approximate of 500 nt. This underscored the indispensability of integrating long-read sequencing technology to expand the ability for reconstructing longer circRNAs, thereby enriching our understanding of circRNA landscapes.

Based on the reconstructed full-length circRNA sequences, we further calculated the orthologous circRNA across diverse species. Here, the MCS score metric (13) was employed to measure the conservation of circRNAs. Similar to circAtlas 2.0, most of the circRNAs were only detected in limited species and tissues (Figure 2E), which is consistent with the high species and tissue specificity of circRNAs (12). We further divided all circRNAs into two categories: species-specific circRNAs (only detected in one species) and orthologous circRNAs (consistently expressed across multiple species). While as expected, species-specific circRNAs dominated most species (Figure 2F), a significant proportion of orthologous circRNAs was observed between human and rhesus, which could be explained by the close relationship between human and rhesus. Taken together, these results demonstrate that circAtlas 3.0 can be applied for exploring the evolution of circRNAs in different vertebrate species.

### Database structure

The circAtlas 3.0 database consisted of 3.18 million consistently named circRNAs, which constituted a comprehensive relationship database for circRNA expression and functional annotation (Figure 2F). For each circRNA, the basic information including circAtlas ID, unified ID, BSJ position, and other attributes was stored in the circRNA table, while supplementary details including full-length isoforms, expression values, functional annotations and conversion to other genome assemblies or circRNA resources were stored as separate tables. In summary, a total of 130 MySQL tables with 441 049 450 entries were generated, resulting in ∼36 GB of MySQL data. Here, this huge dataset provides an important basis for the exploration of circRNA landscape using our updated circAtlas 3.0 database.

### Web interface and applications

**Figure 3.**
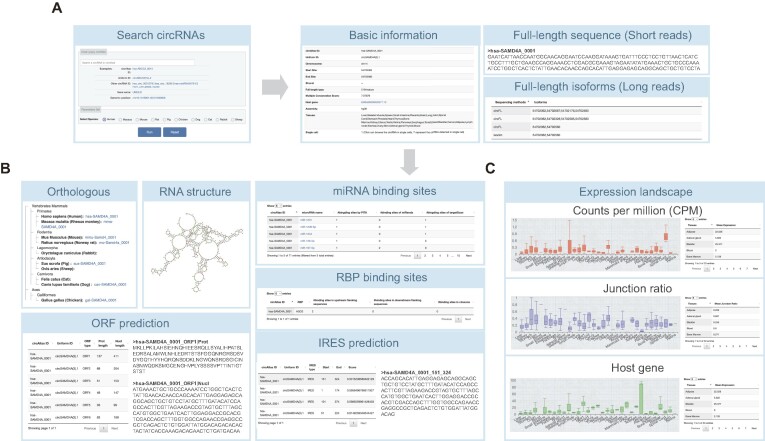
Basic search and analysis functions in circAtlas 3.0. (**A**) Summary of the search function and basic information of circRNAs. (**B**) Summary of the orthologous circRNAs and functional analysis results based on full-length sequences. (**C**) Visualization of the expression levels of circRNAs and their host genes. The distribution of junction ratio in different species is also shown to represent the strength of back-splicing events.

The circAtlas 3.0 database provides user-friendly search and download functionalities, designed to explore the circRNA expression landscape and their biological functions across the 10 collected species. As shown in Figure 3A, users can (i) search for specific circRNAs by circAtlas ID, unified ID, genomic position and host gene name or ID; (ii) browse all circRNAs in specific species and optionally sorted by MCS scores or circRNA length; (iii) select the top 30 highly expressed circRNAs per tissue. After selecting a target circRNA, the basic information will be displayed, including the full-length isoform sequences in both short-read and long-read sequencing datasets.

Based on the full-length sequences, circAtlas 3.0 provides a variety of analysis results (Figure 3B). First, circAtlas 3.0 provides the orthologous circRNAs across various species, the user can click the links of orthologous circRNA, which provides important criteria for selecting species-specific or highly conserved circRNAs. Furthermore, circAtlas 3.0 provides useful functional analysis results, including (i) RNA secondary structure prediction, which provides useful information for linking RNA structure with their biological functions; (ii) miRNA binding sites using three different miRNA prediction software. Each row represents the number of binding sites of specific microRNA; (iii) RBP binding sites, where each row represents the number of binding sites in the circular exons and upstream/downstream flanking regions; (iv) IRES prediction results. Each row indicates the position and sequence of the predicted IRES element and (v) ORF prediction results. The predicted nucleotide and peptide sequences of the ORF are shown. The IRES and ORF prediction results can be retrieved using the circRNA ID on a separate page. Collectively, these analysis results provide insight into the common function of circRNAs as miRNA/RBP sponges or encoding short peptides. Finally, circAtlas 3.0 provides the expression landscape of the target circRNA and its host gene within 33 different tissues (Figure 3C). To explore the splicing strength of back-splicing events, the junction ratio of circRNA is also shown. The change of expression level and junction ratio provides the basis for exploring linear-circular switching and circular transcript usage switching events (30). In summary, the rich information of circAtlas 3.0 provides useful resources for functional prioritization of circRNAs across 10 vertebrate species.

### Integration of other useful resources

**Figure 4.**
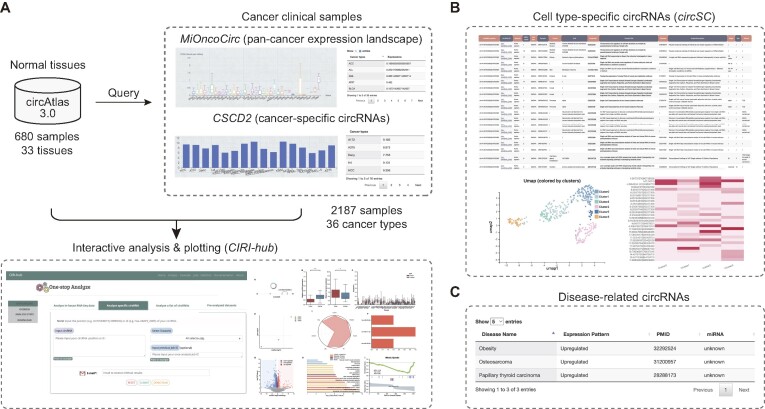
Overview of the integrated links to other resources. (**A**) circAtlas 3.0 provides the circRNA expression landscape in clinical cancer samples (MiOncoCirc & CSCD2), and links to CIRI-hub for interactive analysis and plotting of circRNAs in cancer. (**B**) circAtlas 3.0 provides links of circRNAs detected in full-length single-cell sequencing datasets (circSC). (**C**) circAtlas 3.0 integrates the circad, circR2Disease and circRNADisease databases to provide curated functions of disease-related circRNAs.

The previous circAtlas 2.0 consisted mainly of normal tissues samples. However, since circRNAs have important regulatory roles in disease and cancer, we also integrated the expression landscape of circRNAs in clinical samples across 36 cancer types from the MiOncoCirc (57) and CSCD2 (58) databases (Figure 4A). The expression levels of circRNAs in different cancer types are also presented in the results page. Moreover, we provided a link to CIRI-hub (59), an online interactive analysis platform based on human samples collected in the circAtlas, MiOncoCirc and CSCD2. Additionally, circAtlas 3.0 also included the link to circRNAs in circSC (60), allowing user-friendly analysis of the single-cell level expression landscape of target circRNAs (Figure 4B). Finally, circAtlas 3.0 integrates 2269 latest curated disease-associated circRNAs from the circad (61), circR2Disease (62) and circRNADisease (63) databases (Figure 4C). Taken together, the integration of these online resources provides a useful platform for exploring the biological function of target circRNAs.

### Comparison with other resources

Recent studies have developed several databases to investigate circRNAs in different tissues and species (Supplementary Table S2). For instance, circBase (9) contains 96891 circRNAs from 78 samples across five species, and CIRCpedia v2 (10) consists of 262782 circRNAs from 185 samples from six species. TSCD (64) contains 284 296 circRNAs from 16 adult and 15 fetal human tissues. Notably, most of these databases are limited by the small and outdated number of samples and species, making them difficult to get a comprehensive overview of circRNAs in vertebrates. Among them, our circAtlas 2.0 database (13) provides the largest collection of 1 million circRNAs from 1070 samples, but it is still limited to model species such as humans, rhesus, mouse and rat. In addition, MiOncoCirc (57), CSCD2 (58) and CircRic (65) focus on circRNAs in clinical cancer samples, but lack normal tissues as a control. In this study, we presented the most comprehensive set of circRNAs and their expression and functional profiles from 2674 RNA-seq datasets across 10 species. The new features of circAtlas 3.0 are as follows: (i) the largest collection of over 3.1 million circRNAs from 10 species; (ii) integration of accurate full-length sequences from nanopore long reads; (iii) embedded expression landscape of circRNAs in both normal tissues and cancer samples; (iv) the employment of a standardized nomenclature scheme recently proposed by the circRNA community. Taken together, circAtlas 3.0 provides convenient features for studying the evolution and biological functions of vertebrate circRNAs.

## Discussion

The circAtlas 3.0 provides the largest collection of more than 3.2 million consistently named circRNAs across 10 species and 33 tissue types. The circAtlas 3.0 has two unique features: (i) it is the first database to integrate Illumina and Nanopore circRNA sequencing datasets, providing the full-length sequences and downstream functional analysis results of over 2.5 million circRNAs. The accurate full-length sequence is essential for exploring the biological function of circRNAs; (ii) it is the first database to employ the standardized nomenclature scheme proposed by the circRNA community. The unified naming can ensure clarity and reproducibility between different resources, which eliminates the ambiguous naming and reduces the meaningless effort in converting circRNA IDs between different resources. In addition, circAtlas 3.0 incorporates circRNA expression profiles from clinical cancer samples and provides links to other useful resources such as CIRI-hub, circSC and diverse disease-related databases. These extensive datasets provide an important foundation for in-depth exploration into the roles of circRNAs in cancer and disease.

With the increasing number of long-read circRNA sequencing data, the future version of circAtlas could be enhanced by including assimilating additional long-read sequencing datasets, which will greatly facilitate the identification of longer circRNA isoforms. Furthermore, the inclusion of diverse vertebrate classes will broaden the circRNA landscape in the current databases and help us to understand the evolution of circRNAs. Overall, circAtlas 3.0 provides a useful resource of circRNAs across a variety of species and tissues as well as clinical samples, to improve our understanding of the significance of circRNAs in evolution and disease.

## Supplementary Material

gkad770_Supplemental_File

## Data Availability

The circAtlas 3.0 database is freely available at http://circatlas.biols.ac.cn and https://ngdc.cncb.ac.cn/circatlas. The RNA-seq datasets in circAtlas 3.0 can be accessed in Supplementary Table S1.

## Notes

Present address: Wanying Wu, Department of Computational Molecular Biology, Max Planck Institute for Molecular Genetics, Ihnestraße 63-73, 14195 Berlin, Germany.
