# Supplementary material for: circAtlas 3.0: a gateway to 3 million curated vertebrate circular RNAs based on a standardized nomenclature scheme
Source: Nucleic Acids Res. 2023 Sep 22;52(D1):D52–60. doi: 10.1093/nar/gkad770 (PMC10767913; doi:10.1093/nar/gkad770)
Supplement: gkad770_Supplemental_File [file gkad770_supplemental_file.pdf]

Supplementary Table 1. Summary of updated datasets in the circAtlas 3.0 database

GSE	PMID	Title	Journal	DOI
GSE45326	24344320	Identification of expressed and conserved human noncoding RNAs.	RNA (New York, N.Y.)	10.1261/rna.038927.113
GSE46523	24149843	HAMR: high-throughput annotation of modified ribonucleotides.	RNA (New York, N.Y.)	10.1261/rna.036806.112
GSE56185	24752171	Alternative splicing regulates vesicular trafficking genes in cardiomyocytes during postnatal heart development.	Nature communications	10.1038/ncomms4603
GSE52463	24647608	Transcriptome analysis reveals differential splicing events in IPF lung tissue.	PloS one	10.1371/journal.pone.0092111
GSE53960	24510058	A rat RNA-Seq transcriptomic BodyMap across 11 organs and 4 developmental stages.	Nature communications	10.1038/ncomms4230
GSE53960	25977771	Comprehensive RNA-Seq transcriptomic profiling across 11 organs, 4 ages, and 2 sexes of Fischer 344 rats.	Scientific data	10.1038/sdata.2014.13
GSE60833	27657503	The Impact of Endurance Training on Human Skeletal Muscle Memory, Global Isoform Expression and Novel Transcripts.	PLoS genetics	10.1371/journal.pgen.1006294
GSE60655	25484259	An integrative analysis reveals coordinated reprogramming of the epigenome and the transcriptome in human skeletal muscle after training.	Epigenetics	10.4161/15592294.2014.982445
GSE58608	25016029	The human skeletal muscle transcriptome: sex differences, alternative splicing, and tissue homogeneity assessed with RNA sequencing.	FASEB journal : official publication of the Federation of American Societies for Experimental Biology	10.1096/fj.14-255000
GSE61991	25714049	Neural circular RNAs are derived from synaptic genes and regulated by development and plasticity.	Nature neuroscience	10.1038/nn.3975

GSE64417	26076956	Statistically based splicing detection reveals neural enrichment and tissue-specific induction of circular RNA during human fetal development.	Genome biology	10.1186/s13059-015-0690-5
GSE65926	25921068	Circular RNAs in the Mammalian Brain Are Highly Abundant, Conserved, and Dynamically Expressed.	Molecular cell	10.1016/j.molcel.2015.03.027
GSE71832	28232790	Cortical Morphogenesis during Embryonic Development Is Regulated by miR-34c and miR-204.	Frontiers in molecular neuroscience	10.3389/fnmol.2017.00031
GSE71832	26541409	Spatio-temporal regulation of circular RNA expression during porcine embryonic brain development.	Genome biology	10.1186/s13059-015-0801-3
GSE72879	26546448	Circular RNAs in monkey muscle: age-dependent changes.	Aging	
GSE73763	31552085	Comprehensive Profiles of mRNAs and miRNAs Reveal Molecular Characteristics of Multiple Organ Physiologies and Development in Pigs.	Frontiers in genetics	10.3389/fgene.2019.00756
GSE73763	28575165	Genome-wide profiling of Sus scrofa circular RNAs across nine organs and three developmental stages.	DNA research : an international journal for rapid publication of reports on genes and genomes	10.1093/dnares/dsx022
GSE73763	28233874	Comprehensive analysis of long non-coding RNAs highlights their spatio-temporal expression patterns and evolutional conservation in Sus scrofa.	Scientific reports	10.1038/srep43166
GSE73763	28045116	Comparative analysis of DNA methylome and transcriptome of skeletal muscle in lean-, obese-, and mini-type pigs.	Scientific reports	10.1038/srep39883
GSE74747	26768488	Complex regulation of ADAR-mediated RNA-editing across tissues.	BMC genomics	10.1186/s12864-015-2291-9

GSE77329	27119355	Potential diagnostic and prognostic marker dimethylglycine dehydrogenase (DMGDH) suppresses hepatocellular carcinoma metastasis in vitro and in vivo.	Oncotarget	10.18632/oncotarget.8927
GSE77661	27050392	Circular RNA profiling reveals an abundant circHIPK3 that regulates cell growth by sponging multiple miRNAs.	Nature communications	10.1038/ncomms11215
GSE83115	27329541	Identification of Tissue-Specific Protein-Coding and Noncoding Transcripts across 14 Human Tissues Using RNA-seq.	Scientific reports	10.1038/srep28400
GSE89355	30791438	Circular RNA circHIPK3 Promotes the Proliferation and Differentiation of Chicken Myoblast Cells by Sponging miR-30a-3p.	Cells	10.3390/cells8020177
GSE89355	29868120	Circular RNA circSVIL Promotes Myoblast Proliferation and Differentiation by Sponging miR-203 in Chicken.	Frontiers in genetics	10.3389/fgene.2018.00172
GSE91059	28286579	Brain xanthophyll content and exploratory gene expression analysis: subspecies differences in rhesus macaque.	Genes & nutrition	10.1186/s12263-017-0557-3
GSE106381	29538394	Long non-coding RNAs and mRNAs profiling during spleen development in pig.	PloS one	10.1371/journal.pone.0193552
GSE107376	29545200	Alterations in Placental Gene Expression of Pregnant Women with Chronic Chagas Disease.	The American journal of pathology	10.1016/j.ajpath.2018.02.011
GSE109082	29335024	Sex differences in the late first trimester human placenta transcriptome.	Biology of sex differences	10.1186/s13293-018-0165-y
GSE120795	31015567	Atlas of RNA sequencing profiles for normal human tissues.	Scientific data	10.1038/s41597-019-0043-4
GSE128537	31980617	Transcriptomic and open chromatin atlas of high-resolution anatomical regions in the rhesus macaque brain.	Nature communications	10.1038/s41467-020-14368-z
GSE138734	34140680	The RNA Atlas expands the catalog of human non-coding RNAs.	Nature biotechnology	10.1038/s41587-021-00936-1

GSE154616	34556162	SRCP: a comprehensive pipeline for accurate annotation and quantification of circRNAs.	Genome biology	10.1186/s13059-021-02497-7
GSE175633	34831115	Transcriptome Analysis of Testicular Aging in Mice.	Cells	10.3390/cells10112895
GSE77276	28194035	Recurrently deregulated lncRNAs in hepatocellular carcinoma	Nature communications	10.1038/ncomms14421
ERP014416		J-Line Chicken 21 Tissue RNA Expression		
GSE77314	27119355	Potential diagnostic and prognostic marker dimethylglycine dehydrogenase (DMGDH) suppresses hepatocellular carcinoma metastasis in vitro and in vivo	Oncotarget	10.18632/oncotarget.8927
GSE162140	34140474	A pig BodyMap transcriptome reveals diverse tissue physiologies and evolutionary dynamics of transcription	Nature communications	10.1038/s41467-021-23560-8
GSE37909		RNA-seq from ENCODE/Caltech (Mouse)		
GSE49906	24752171	Alternative splicing regulates vesicular trafficking genes in cardiomyocytes during postnatal heart development	Nature communications	10.1038/ncomms4603
GSE64283	26076956	Statistically based splicing detection reveals neural enrichment and tissue-specific induction of circular RNA during human fetal development	Genome biology	10.1186/s13059-015-0690-5
SRP007584	22009989	Fusobacterium nucleatum infection is prevalent in human colorectal carcinoma	Genome Research	10.1101/gr.126516.111
PRJCA000751	30893614	Expanded Expression Landscape and Prioritization of Circular RNAs in Mammals	Cell reports	10.1016/j.celrep.2019.02.078
Retinas	30874468	Circular RNAs in human and vertebrate neural retinas	RNA Biology	10.1080/15476286.2019.1591034
PRJNA475651	30660194	Reconstruction of full-length circular RNAs enables isoform-level quantification	Genome medicine	10.1186/s13073-019-0614-1
CRA003317	33707777	Comprehensive profiling of circular RNAs with nanopore sequencing and CIRI-long	Nature Biotechnology	10.1038/s41587-021-00842-6

PRJNA722575	34647522	circFL-seq reveals full-length circular RNAs with rolling circular reverse transcription and nanopore sequencing	Elife	
GSE141693	33436621	isoCirc catalogs full-length circular RNA isoforms in human transcriptomes.	Nature communications	10.1038/s41467-020-20459-8
GSE223104	37402719	Defining the landscape of circular RNAs in neuroblastoma unveils a global suppressive function of MYCN	Nature communications	10.1038/s41467-023-38747-4

Supplementary Table 2. Comparison of circAtlas 3.0 and other databases.

Database	Publication	#circRNAs	#Samples	#Species	Sample source
**circAtlas 3.0**	**This study (2023)**	**3,179,560**	**2,674**	**10**	**Normal**
circAtlas 2.0	*Genome Biology* (2020)	1,007,087	1,070	6	Normal
circBank	*RNA Biology* (2019)	140,790	78	1	Not specified
circPedia v2	*Genomics Proteomics & Bioinformatics* (2018)	262,782	185	6	Not specified
circRNADb	*Scientific Reports* (2016)	32,914	11	1	Not specified
circBase	*RNA Biology* (2014)	96,891	78	5	Not specified
circNet 2.0	*Nucleic Acids Research* (2021)	289,303	2,732	1	Cancer
MiOncoCirc	*Cell* (2019)	227,056	2,093	1	Cancer
CSCD2	*Nucleic Acids Research* (2021)	1,013,461*	1,113	1	Cancer
CircRic	*Genome Medicine* (2019)	92,589	935	1	Cancer

*Number of cancer circRNAs available in CSCD2.
